# Mineral and Thermal Springs of the United States

**Published:** 1855-06

**Authors:** John Bell


### *Mineral and Thermal Springs of the United States.*

By John Bell, M.D.

While engaged in preparing my full and elaborate work on Mineral and Thermal Springs, which, I regret to say, will not make its appearance this year, I have thought that a brief notice of those of the United States would be acceptable at this time. It will, at least, serve to give a wider range of selection for those who are yet undecided as to the spot which they may visit in the dog-days.

I shall not begin far "down east;" and shall, therefore, first notice the *Berkshire Soda Spring*, within three miles of the beautiful village of Great Barrington, Berkshire county, Massachusetts, through which four daily trains of cars pass. Great Barrington is 28 miles from the Hudson river and the city of Hudson. The chief mineral impregnation of this water is in the soda, combined with carbonic acid and chlorine, and a little alum.

*Oak Orchard Acid Mineral Springs.*—Under this title we meet with a very active water, holding in solution, sulphates of magnesia, alumina and iron, with other salts, and an excess of sulphuric acid, constituting what is called Alum Water of some of the Virginia Springs. It is an active tonic and astringent.

These springs are about six and a half miles south of the village of Medina, on the Erie canal, and eighteen south-east from Lockport, Genesee County, New York.

*Avon Sulphur Springs.*—The waters of these springs are among the strongest of the sulphureous class, and contain also sulphate of soda and other salts, which render them purgative. In one of them (in the Sylvan group) iodine has been detected.

Avon is on the eastern bank of the Genesee river, in Livingston County, New York, eighteen miles from Rochester. The springs were known to the Indians, who resorted to them for the cure of diseases,—a reputation well deserved and sustained to the present day.

*Saratoga Springs.*—These springs are deservedly celebrated, and nowhere can be found mineral waters of the carbonated saline class at once so pleasant and endowed with so much medicinal activity. Then, in addition to the chief fountain, there are iodine and chalybeate springs, so that all desirable variety is furnished for the relief or cure of a large circle of diseases. We need not give here an analysis of these waters ; but refer to the treatises of Steele, North, and Allen on this point, and on their medicinal uses.

*Lebanon Spring.*—This is ranked, on the score of temperature, among the Thermal springs, and of gaseous impregnation, among the nitrogen ones. The water is constantly at 73° F., while that of the other springs in the county (Rensellær) is 52° F. Its saline impregnation is very slight, being only a grain and a quarter in the pint. The temperature is such as to render the Lebanon water a delightful bath. So copious is the supply that not only is there an abundance for all the baths, but also enough to turn two or three mills erected within a short distance. These are kept running during even the severity of the winter.

*Albany Mineral Springs.*—They are represented to be very similar to those of Saratoga and Balston ; but they contain a smaller proportion of carbonic acid gas.

*Canandaigua Sulphureous Springs.*—These are eight miles north-west of the town. They gush from a hill in so copious a stream as to turn a mill.

This class of waters is very numerous in the State of New York. At *Buffalo* there is one of considerable strength. The water contains, in addition, carbonates of lime, magnesia and soda, and sulphate of lime.

*Sharon Springs.*—These sulphureous waters almost rival those of Saratoga, in the reputation which they have acquired for the cure of various chronic maladies ; while the spot itself has superior natural beauties. One of the springs, the Magnesian, has a greater proportion of saline contents, and a greater tendency to act on the bowels than the other ; and hence it is used in cases in which the exciting effects of the latter, or White Sulphur as it is called, would be injurious.

In addition to the well known Brine Springs and Salines in New York, there are, also, many medicinal saline springs in the districts in which the former prevail.

*Byron Acid Spring.*—In the salt group of Onondaga, in a portion of the Silurian system of the State, we meet with this spring, the water of which contains an excess of sulphuric acid, by which and its other contents it ranks among the Alum Springs.

*Sulphureous Springs of Vermont.*—Among these we may enumerate the *Highgate* springs, within 12 miles of the steamboat landing at St. Alban's Bay; also those of Newbury 27 miles east of Montpelier and 47 north-east from Windsor; and those of Alburgh.

*Bennington Thermal Spring.*—It is thus designated by Professor Hitchcock, who does not, however, give its temperature. It emits both nitrogen and oxygen gases. The water is abundant enough to turn the machinery of a powder mill.

*Schooley's Mountain Springs.*—These are the most noted and most frequented of the mineral springs of New Jersey. The water 50° F. is a mild saline chalybeate, the virtues of which are greatly aided by the cool and invigorating air of this spot.

*Bedford Springs.*—These rank foremost in Pennsylvania, on account of their mineral properties and medicinal effects, and their mountain elevation and surrounded scenery. They are two miles from the town of Bedford, and less than 200 miles from Philadelphia and not 100 from Pittsburg: they are 130 miles from Baltimore, and the same distance from Washington. The water of the chief spring is a saline chalybeate. Others have received the designations of *Limestone, Sweet, Sulphur* and *Chalybeate.* The most active ingredients in the first or main spring are sulphate of magnesia and carbonate of iron. The temperature of the water is 55° F., which must be somewhat higher than the common springs of this region.

The Bedford water has acquired deserved celebrity in indigestion and chronic diarrhœa and dysentery, and in renal diseases, in which the inflammation or inflammatory excitement has subsided, and there remains an atonic and enfeebled condition of the organs. In uterine and in cutaneous diseases which have reached this stage, and have assumed a chronic character, it is, also, of decided benefit. The gouty and rheumatic in whom there is no plethora or cerebral determination, have also reason to speak well of this water.

What with the invigorating effects of the water, and of the mountain air and mountain walks, the visitors must be supposed to have a keen appetite, and to require a bountifully supplied table; the more so as the majority at a watering place do not rank themselves among the class of invalids, and have to rely on something more than drinking the water or bathing, to keep up their strength and spirits. Of the way in which these things are managed at Bedford I cannot speak from personal experience.

Within a day's ride of Bedford are the little town of Bath, in Virginia, and its thermal spring, of which further mention will soon be made.

*York Springs.*—One of them is saline, the other a strong chalybeate. The water of the first is said to be diuretic and mildly cathartic. This place is readily reached by railroad from Philadelphia and Baltimore. It is in Adams County; "two to four hours ride of Gettysburg, Carlisle, Harrisburg and Hanover."

*Perry County Springs.*—These, erroneously called "Warm," are so far thermal as to be, probably, fifteen or eighteen degrees higher than the common spring of the country; and hence the water would furnish a pleasantly cool, approaching to a temperate bath. They are fourteen miles from Harrisburg and the same distance from Duncannon, on the Central Railroad, and at the foot of Pisgah mountain, in a district which allows of fine drives and rides.

*Carlisle Springs.*—The water of these springs is a mild sulphureous one. They are within a short distance of the town of Carlisle, which is traversed by the railroad from Philadelphia to Chambersburgh.

*Doubling Gap Sulphureous and Chalybeate Springs.*—They are situated in a gap formed by the doubling of the Kittitany or North Mountain, about thirty miles south-west of Harrisburg. The Cumberland Valley Railrord passes through Neuville, distant eight miles from the Springs, to which visiters are taken by stages.

*Bath Chalybeate Springs.*—At one time these springs used to be visited by many of the citizens of Philadelphia, on account, in good part, of ready access to them. They are within a short distance of Bristol, on the Delaware.

Besides these Mineral Springs of Pennsylvania now enumerated, there are cold springs of pure water, which, owing to their situation in healthy and romantic districts of country and the facilities furnished for cold bathing, have acquired deserved vogue. Of these I shall notice—

*The Yellow Springs.*—They are in Chester County, thirty miles from Philadelphia; and are reached from this city by railroad and stages. The view of the surrounding country is fine, and facilities are given for excursions in different directions. In addition to the natural baths of the temperature of the chief spring, which is 53° F., warm ones are also obtainable. The house is well kept, and the table every way good.

*The Ephrata Mountain Springs.*—These springs, situated 13 miles north of Lancaster, are resorted to by large numbers of people every year. The scenery, the grounds around, the hotel accommodations and the means for procuring baths of various temperatures, present strong inducements to visit this spot.

*Caledonia Springs.*—To the inhabitants of the city who have suffered from the wear and tear of business, to the invalid slowly recovering from disease, to those who have become weakened and exhausted in the giddy round of pleasure, and to all who would like to see Nature in her nobler aspects, this spot is eminently inviting. New scenes and new associations of a genial and abiding character are opened to us; and, for a time at least, we feel ourselves relieved from leaden cares, and enjoy a sense of unwonted freedom. While nature has been so bountiful in her mountain and woodland views, her pure and copious springs and streams, and her vivifying and exhilarating air, art has also contributed its share to the comfort of the visitors to these springs, who are received in a new, spacious and well ordered hotel, are comfortably lodged, and sit down to a table every way well supplied—the viands good, abundant, and prepared with due culinary skill.

The Caledonia, long known as Sweeny's Cold Springs, have enjoyed, during many years past, quite a reputation, when used as a bath, for the cure of chronic rheumatism and various other diseases in which there is a blending of still remaining febrile heat and irritation with debility. Warm baths are, also, always to be had.

The springs are situated on the South Mountain, which rises in the rear of the hotel, and in front they command an extensive view of alternate woods and fields, terminated by a semicircular sweep of the North Mountain.

Without meaning to undervalue the efficacy of mineral waters, the writer can recommend invalids, or the weak who wish to become stronger, to make the regular drinking of the singularly pure water of one of the springs, before breakfast and before dinner, a part of the pleasant regimen of good eating, sound sleeping, and varied exercises which he will enjoy at this favored spot.

The Caledonia Springs are fifteen miles from Chambersburg; the greater part of the road being the turnpike which unites this town with Gettysburg. Visitors, on their arrival by railroad from Philadelphia or Baltimore, are taken out immediately in omnibuses or other vehicles from Chambersburg to the springs, which they reach on the evening of the same day of their leaving either of the cities just named.

Virginia is peculiarly rich in mineral springs, and until the acquisition of California and New Mexico, had more thermal ones than any other portion of the United States. Beginning at the North and advancing to the South, we first meet with—

*The Bath* (Berkeley county) *Spring.*—This, like the Sweet Springs, is a mild carbonated or acidulous thermal water, of the temperature of 73° F., the same as that which in England, by a strange blunder, is called Bristol Hot Well. It has been very serviceable in a variety of chronic diseases, when used as a bath. Persons who went there crippled with chronic rheumatism have come away quite restored to the free use of their limbs, and as agile in all their movements as the country people around. The internal use of the water merits more attention than it has generally received, especially in atonic and irritable dyspepsia and chronic bowel diseases.

Bath is within a short distance of the Baltimore and Ohio railroad, if we are not mistaken; and, as already stated, it is not far from Bedford, Pa.

*Shannondale Saline Springs.*—These springs are within a few miles of Charlestown, Jefferson county, through which the railroad from Harper's Ferry to Winchester passes. The water acts as a mild aperient and diuretic, and it is adapted, in consequence, to a large circle of diseases. The springs are near the banks of the Shenandoah, the sound of whose waters is heard with an agreeable effect at the Hotel on the hill, where the visitors are quartered. There are few spots in the Union which present so many natural advantages and capabilities for extended walks, gardens and groves as Shannondale.

*Jordan's White Sulphur Springs.*—They are about five or six miles from Winchester, and four miles from the railroad between this town and Harper's Ferry. Passengers leaving Baltimore in the morning will reach the springs between three and four o'clock in the afternoon. The waters are serviceable in chronic dyspepsia, with a torpid state of the liver, chronic rheumatism, cutaneous affections, and the debility left by fevers.

Many of the visitors to the upper or mountain springs in the south western part of the State, spend a few days here on their return to the north ; and those from the Eastern Shore and Northern Neck linger late in the season, until it is safe for them to go home, with a prospect of escaping an attack of their endemic fevers.

*Capon Springs.*—These have come greatly into vogue, and it would seem not without good reason. The water is beneficial in certain forms of dyspepsia, and in renal affections, especially, as we are told, in the lithic acid diathesis. The arrangements for cold bathing are on a large scale, and the baths of a superior kind ; warm bathing can also be enjoyed by those who claim it, either as a hygienic agent, or a remedy for disease. Mountain air largely inhaled gives a keener relish for the mountain mutton, of which the lovers of good cheer speak so highly at this place. A hotel of the first class has been erected, and furnishes good quarters to a large number of visitors. Not a few have their own houses or cabins.

The Capon Springs are about 30 miles from Winchester. Whether the fear of diminishing the reputation of the water as a medicinal agent, by showing how very slight is its medicinal impregnation, or owing to the indolence of the parties more directly interested in the question, we cannot say ; but, as yet, there has been no analysis made, or, at any rate, reported, which has come under my notice.

*Fauquier or Warrenton White Sulphur Springs.*—They derive the first name from the county, the second from the town in which they are situated. They are within a few miles of the railroad from Alexandria, and at the same distance from the one to Staunton, which traverses the Valley; and also by stages from Winchester.

The waters are of a mild sulphureous nature, but of the proportion of their gaseous and solid contents we are ignorant. Numerous cases are recorded of their efficacy in dyspepsia, chronic diarrhœa, and chronic rheumatism, also in renal affections and disorders of females; but without very minute specification of the organic lesions in the latter.

Attractions superior even to those of the waters are offered to the crowd of visitors, in a noble mansion, as a hotel, and extensive and tastefully arranged grounds, ornamented with shrubberies and parterres. In addition to the main building, a pavilion, which has a portico on its western front, commanding a view of the lawn, and an extensive picturesque region beyond, there are several brick buildings of a large size. Ample provision is made for all the varieties of bathing.

After traversing the beautiful and fertile region known as the Valley, between the Blue Ridge and the Alleghany mountains, which begins at Harper's Ferry and ends at the Natural Bridge, south of Lexington, we find ourselves in the vicinity of the celebrated "Virginia Springs." We know of no place in the world, of the same extent, which is marked by such a number and variety of mineral and chemical springs as the one now under notice. It possesses, at the same time, the advantages of a fine climate and scenery of a highly diversified character. The company at the several springs, free from aristocratic pretentions and ridiculous attempts at exclusiveness, always exhibits a large share of intelligence, good taste and sociability. The infusion of the Virginian and Southern element—a frank, cordial address and good humor—adds not a little to the pleasures of the Northern visitors, who, with excellent intentions, are not remarkable for that ease of manner and confiding speech which invite intimacy.

Leaving Staunton with a design to visit the "Springs," we shape our course west, and at a distance of 45 miles in this direction we reach

*The Bath Alum Springs.*—They are on the main road from Richmond to Guyandotte, on the Ohio river, at the eastern base of the Warm Spring mountain, and a few miles east of the Springs themselves.

An analysis of the water of one of the Bath Alum Springs, that most used, by Dr. Hayes, of Boston, shows it to contain, in a gallon, nearly fifty-five grains of saline substances, and of carbonic and sulphuric acid. Those most active, are the salts of iron, and alumina, and on them and the free sulphuric acid, the sensible properties and curative powers of these waters in a great measure depend. Being a strong tonic and astringent, it is easy to indicate a number of diseases in which they must be of service, and, already, experience has proved in many respects what *à priori* reasoning would have suggested respecting their beneficial operation. We may specify, as first on the list, chronic affections of the digestive mucous membranes, including those of the throat, stomach and bowels, and marked by feebleness or imperfection of function and morbid secretions. Similar praise may be extended to it in chronic diseases of the urinary and generative organs, and cutaneous diseases, chronic ulcers, simulating cancers, and scrofula have been greatly relieved, and in some cases entirely cured by the methodical drinking of these waters—a result quickened by their external application to the sore or tumor.

The accommodations for the reception and entertainment of visitors are represented to be of a superior kind at these springs.

*Rockbridge Alum Springs.*—If, in place of turning off west from Staunton, we were to go further south, to Lexington and the Natural Bridge, and then visit the Warm Springs, we should meet with these alum springs on the road. They are 17 miles from the town just mentioned, and 22 from the Warm Springs, in a valley between the North Mountain on the East, and the Mill Mountain on the West.

The composition of the waters of these Springs, as ascertained by Dr. Hayes, is similar to that of the Bath Alum Waters, and both of them resemble those of the Oak Orchard Springs, and the Acid Byron one in New York. The Rockbridge water is stronger in the proportion of free sulphuric acid and the sulphate of alumina, but contains less iron than the Bath waters. Its use is applicable to the same diseases in which the other is beneficial, with the modifications required by the differences in chemical composition just now stated.

(To be continued.)

